# Head-to-Head Comparison of Humoral Immune Responses to Vi Capsular Polysaccharide and *Salmonella* Typhi Ty21a Typhoid Vaccines–A Randomized Trial

**DOI:** 10.1371/journal.pone.0060583

**Published:** 2013-04-08

**Authors:** Anu Kantele, Sari H. Pakkanen, Riitta Karttunen, Jussi M. Kantele

**Affiliations:** 1 Department of Medicine, Division of Infectious Diseases, Helsinki University Central Hospital, Helsinki, Finland; 2 Department of Bacteriology and Immunology, Haartman Institute, University of Helsinki, Helsinki, Finland; 3 Institute of Clinical Medicine, Department of Medicine, University of Helsinki, Helsinki, Finland; 4 Division of Clinical Microbiology, Department of Virology and Immunology, Helsinki University Hospital, Helsinki, Finland; 5 Department of Medical Microbiology and Immunology, University of Turku, Turku, Finland; University Medical Center Utrecht, The Netherlands

## Abstract

**Background:**

The two typhoid vaccines, the parenteral Vi capsular polysaccharide and the oral live whole-cell *Salmonella* Typhi Ty21a vaccine, provide similar levels of protection in field trials. Sharing no antigens, they are thought to confer protection by different mechanisms. This is the first head-to-head study to compare the humoral immune responses to these two vaccines.

**Methods:**

50 age- and gender-matched volunteers were immunized, 25 with the Vi and 25 with the Ty21a vaccine. Circulating plasmablasts reactive with whole-cell *Salmonella* Typhi or one of the typhoidal antigenic structures, Vi, O-9,12, and H-d antigens, were identified as antibody-secreting cells (ASC) with ELISPOT. Homing receptor (HR) expressions were determined. These results were compared with ASC in four patients with typhoid fever. Antibodies to *S.* Typhi lipopolysaccharides were assessed in cultures of ALS (antibodies in lymphocyte supernatants) and in serum with ELISA.

**Results:**

In 49 out of 50 vaccinees, no typhoid-specific plasmablasts were seen before vaccination. On day 7, response to Vi antigen was mounted in 24/25 volunteers in the Vi, and none in the Ty21a group; response to *S.* Typhi and O-9,12 was mounted in 49/50 vaccinees; and to H-d in 3/50. The numbers of typhoid-specific plasmablasts (total of ASC to Vi, O-9,12 and H-d antigens) proved equal in the vaccination groups. The HR expressions indicated a mainly systemic homing in the Vi and intestinal in the Ty21a group, the latter resembling that in natural infection. Plasmablasts proved more sensitive than serum and ALS in assessing the immune response.

**Conclusions:**

The typhoid-specific humoral responses to Vi and Ty21a vaccines are similar in magnitude, but differ in expected localization and antigen-specificity. The unforeseen O antigen-specific response in the Vi group is probably due to lipopolysaccharide contaminating the vaccine preparation. Only the response to Ty21a vaccine was found to imitate that in natural infection.

**Trial Registration:**

Current Controlled Trials Ltd. c/o BioMed Central ISRCTN68125331

## Introduction

Typhoid fever continues to comprise a significant health problem in developing countries, posing a risk for travellers as well [1], [2]. Two vaccines are available for clinical use: the parenteral Vi capsular polysaccharide vaccine, and the oral live *Salmonella* Typhi Ty21a vaccine [1]–[4]. Both have proved immunogenic, providing similar levels of protection against typhoid fever in clinical trials [2]–[4], yet there are no head-to-head studies comparing their immunogenicity and protective efficacy. As the two vaccines do not share antigens, the immune mechanisms underlying their protective efficacy have been considered entirely different [2].

The Vi antigen is thought to be one of the major virulence factors of *S.* Typhi [5]. Vaccines based on Vi capsular polysaccharide have been purified from strain *S.* Typhi Ty2. These parenterally administered vaccines have conferred a protection of 55–68% [4], [6]–[8] while causing only a low rate of adverse effects [9]. The vaccine has been considered to contain solely Vi polysaccharide. Accordingly, the protection has been thought to be mediated on serum Vi-specific antibodies [2].

Oral *S.* Typhi Ty21a vaccine is a live, attenuated whole cell preparation containing all other typhoidal structures but the Vi polysaccharide. It has conferred 51–69% protection in field trials [4], [10]–[12]. The immune mechanisms contributing to protection have been considered to include both humoral and cell-mediated immune mechanisms [13]–[19], the route of the antigen encounter and the mode of action imitating that of a natural infection.

An immune response to any vaccine begins with the appearance of antigen-specific plasmablasts in the circulation in humans [13], [16], [20]–[23]. These represent the effector cells of humoral immunity being distributed to their final effector sites. While the blood flow takes these plasmablasts with equal efficacy everywhere in the body, they can cross the endothelial lining and enter the various tissues only if they carry the types of homing receptors (HR) and chemokine receptors required for homing to that particular tissue [24]. The HR and chemokine receptors expressions can be utilized in the evaluation of the localization of an immune response [22], [25]–[29]. The α_4_β_7_ -integrin has been recognized as the HR guiding the cells to home to the intestine [30], and L-selectin as the HR mainly mediating more systemic homing [31]. An intestinal homing profile after Ty21a vaccination has been recorded in several studies [22], [25]–[28]. We are not aware of any studies characterizing circulating plasmablasts or their homing profile after Vi vaccination.

We conducted a head-to-head study with age- and gender-matched volunteers to compare the magnitude, antigen-specificity and localization of humoral immune responses to the two vaccines. These preparations are not considered to share any structures in common, thus our initial hypothesis was that the plasmablast responses would be targeted against different antigens, yet we wanted to compare the number of plasmablasts and the localization of the response as evaluated by the expression of homing receptors on the plasmablasts. In addition to getting answers to our initial questions, we also found that the antigen-specificities in the plasmablast responses did not differ as much as had been thought.

## Materials and Methods

### Ethics Statement

The study protocol was approved by the ethics committee of the Helsinki University Central Hospital and the Finnish Medicines Agency, and registered in the International Standard Randomised Controlled Trial Number Register (ISRCTN68125331). Written informed consent was obtained from all study subjects.

### Study Design

The protocol for this trial and supporting CONSORT checklist are available as supporting information; see Checklist S1 and Protocol S1.

**Figure 1:**
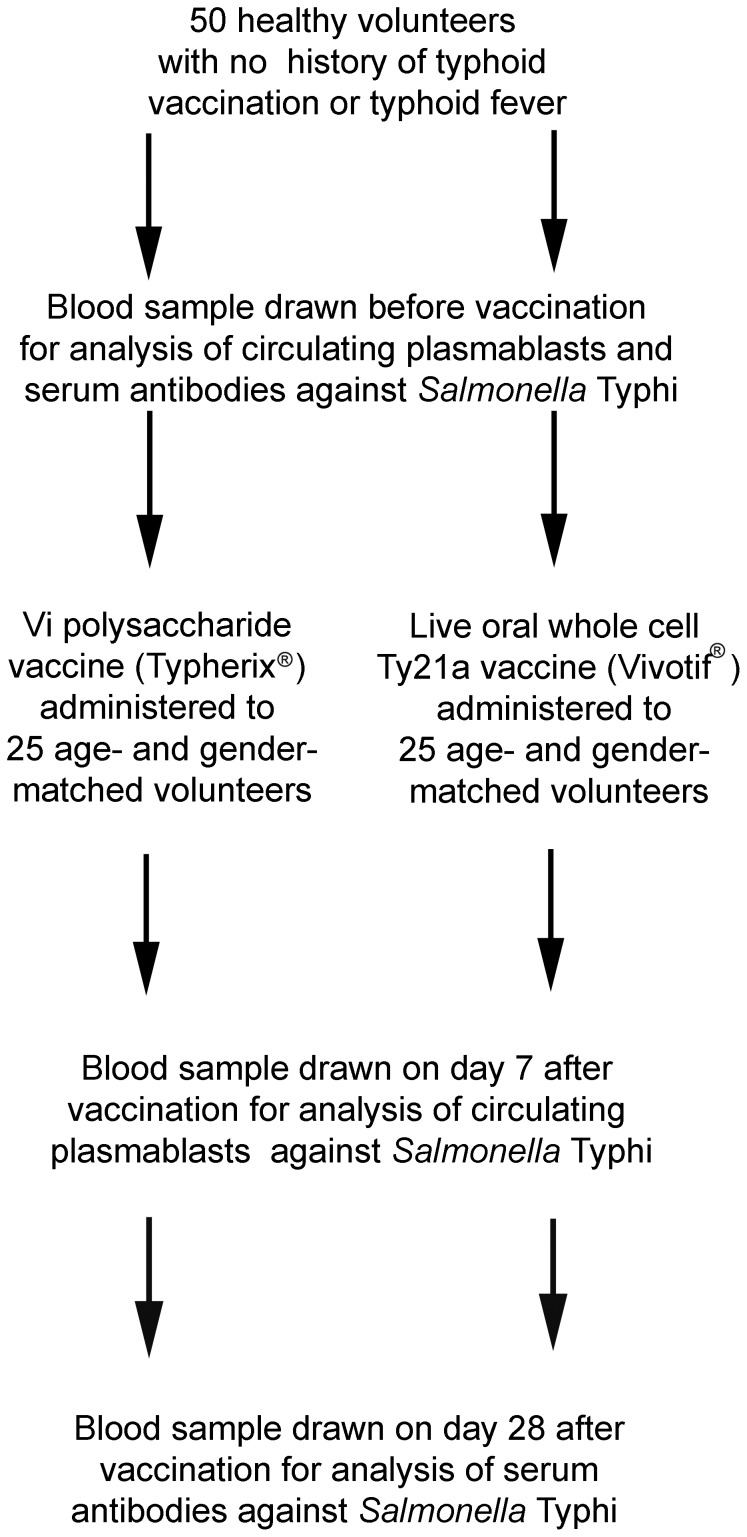
Flow diagram of the study: study groups and timing of samples.

Volunteers vaccinated with Vi and Ty21a vaccines were examined for circulating plasmablasts reactive with strains expressing various typhoidal antigens. The data were compared to those for patients with typhoid fever whose data have been published in our previous studies [32], [33]. Plasmablasts were identified by enzyme-linked immunospot assay (ELISPOT) as antigen-specific antibody-secreting cells (ASC). The homing potentials of *S.* Typhi- specific plasmablasts were characterized by combining immunomagnetic cell sorting with the ELISPOT. Levels of specific antibodies were determined by ELISA in the serum and ALS (antibodies in lymphocyte supernatants) samples of the vaccinees (Figure 1). The study was conducted in Helsinki University Central Hospital and at the Haartman Institute, University of Helsinki between December 2009 and October 2010.

Notably, this report presents only a part of the results of the study protocol provided as a supplementary material. Two recent reports included parts of the data on the same project [32], [33].

### Volunteers, Vaccinations and Samples

The volunteers enrolled were fifty age- and gender-matched healthy Finnish-born volunteers (both groups comprised 17 females, 8 males, all aged 22–62, mean 32 years) (Table 1) with no history of enteric fever or typhoid vaccination, the numbers of volunteers were evaluated in pre-study power calculations based on responses in pilot experiments. Twenty-five of them received the parenteral Vi capsular polysaccharide vaccine (Typherix®, GlaxoSmithKline Biologicals s.a., Rixensart, Belgium, lots ATYPB084BC and ATYPB096AF), and 25 were administered the oral vaccine containing ≥2×10^9^ live *Salmonella* Typhi Ty21a bacteria (Vivotif® Crucell NV, Leiden, The Netherlands, lot 3001777) The amount of contaminating endotoxin in the Typherix vaccine preparation was 27.00 EU per dose for lot ATYPB084BC and 13.30 EU per dose for lot ATYPB096AF (data provided by the manufacturer). The vaccine type given to the first volunteer of each age was determined by raffle and the second, age- and gender-matched subject, was given the other vaccine type. Notably, the results of the assays on the coded ELISPOT plates were read by a blinded person with no knowledge about the types of vaccine given in the various cases, or which plates represented age- and gender-matched pairs. The Vi vaccine was injected with a 25 mm needle as one 0.5 ml intramuscular dose in the left arm on day 0. The Ty21a vaccine was given one capsule on each of days 0, 2, and 4, as recommended by the manufacturer. The data of *S*. Typhi and H-d-specific ASC in volunteers immunized with the Ty21a has been included as a part of a larger recently published set of data [32], [33], where no age- and gender-matched controls were included, while the data on the O-9,12-specific responses have not been presented until now. Both vaccines proved well-tolerated. The adverse effects are described in Table 1. There were no drop-outs in the study.

**Table 1:** Baseline characteristics and adverse effects reported in 50 volunteers immunized with typhoid fever vaccines.

	Vi vaccine	Ty21a vaccine
n	25	25
mean age (range)	32 (22–62)	32 (22–62)
female	17	17
male	8	8
adverse effects		
fever	1	–
pain at the injection site	2	–
constipation	1	–
loose stools	1	–
stomach ache	–	2
tiredness	–	1

The typhoid fever patients have been described earlier [32], [33]: two were Finnish-born travellers returning from Central-America and India, the third was a Sri Lankan applying for asylum, and the fourth a Nepalese immigrant with an infection relapse one month after the first episode. *S*. Typhi strains of the patients were all Vi positive. The data on homing profiles in the patients with primary infection have been reported before, pooled with data from a paratyphoid fever patient [32], [33]; data on the homing profiles of the patient with relapse have not been presented until now.

In our previous studies on mucosal [13], [19]–[21] and parenteral [19] immunization, ASC have been found to appear transiently in the circulation on day 2–3, peaking on day 7 [13], [19], [20]. Accordingly, blood samples were drawn before vaccination and 7 days after it (ELISPOT and ALS), or 7–10 days after the onset of infection symptoms (ELISPOT). Serum samples were collected before and 28 days after vaccination.

### Antigens for ELISPOT

Vi antigen and four whole-cell bacterial strains were used as antigens in the ELISPOT assay (Table 2). As Vi antigen we applied the actual Vi vaccine preparation (Typherix®) at a concentration of 10 µg/ml PBS for coating. The bacterial strains were from the collection of the National Institute for Health and Welfare, Helsinki, Finland. They were chosen to cover selected parts of *S.* Typhi: *Salmonella* Typhi (all antigens), *Salmonella* strain SL2404 (O-9 and 12 antigens), *Salmonella* Egusi (H-d antigen) and *Yersinia enterocolitica* (no antigens in common; negative control). The expressions of the various typhoidal antigens were confirmed in the reference laboratory according to standard methods. The preparation of these strains for use as antigens has been described earlier [32].

**Table 2:** Descriptions of bacterial strains, magnitude of the plasmablast responses to various antigens and results of statistical comparisons in the Vi group.

Antigens, their origin, content of typhoidal antigens and magnitude of the response in the Vi-vaccinated volunteers						Comparison with				
Bacterial strain	Strain/preparation	Vi antigen	O-antigen 9 and 12	H-d antigen	Mean (95%CI)	Vi antigen	O-9,12	H-d	Neg. control	*S.* Typhi
**Vi antigen**	Vi vaccine preparation	+	−	–	107 (51–164)					
**Recombinant ** ***S.*** ** Typhimurium (O-9,12)**	SL2404	−	+	−	41 (18–64)	*				
***S.*** ** Egusi (H-d)**	RHS6854	−	−	+	1 (0–1)	***	***			
***Yersinia enterocolitica*** **(Neg. control)**	RHI4823	−	−	−	1 (0–2)	***	***	NS		
***S.*** ** Typhi**	Vsa61	+	+	+	101 (50–152)	NS	***	***	***	
**Sum of typhoid antigens (Vi+O-9,12+ H-d)**	Value calculated	+	+	+	149 (81–217)	***	***	***	***	***

The bacterial strains used in the ELISPOT assay, the antigens shared with *S.* Typhi by each strain, the number of plasmablasts (ASC/10^6^ PBMC) reactive with the strains (means and 95% confidence intervals), and statistical comparison (Bonferroni-corrected Wilcoxon signed rank test) between the responses in 25 volunteers vaccinated with the Vi capsular polysaccharide vaccine one week earlier. Significant differences are indicated with asterisks (***p<0.001; **0.001<p<0.01; *0.01<p<0.05), NS = not significant.

### Isolation of Peripheral Blood Mononuclear Cells (PBMC)

PBMC were separated using Ficoll-Paque centrifugation as described earlier [13].

### Separation of the Receptor-negative and -positive Cell Populations

The expressions of HR on *S.* Typhi-specific ASC were explored in seven vaccinees in the Vi group and compared to those of nine vaccinees in the Ty21a group reported earlier [32]. Separation of the cells into HR-positive and -negative populations has been described earlier [23], [25]–[27], [29]. Briefly, aliquots of cell suspensions were incubated with monoclonal antibodies against α_4_β_7_ (ACT-1, Millennium Pharmaceuticals, Cambridge, MA), L-selectin (Leu 8, Becton Dickinson, Erenbodegem-Aalst, Belgium), or CLA (HECA-452, a gift from Dr. Sirpa Jalkanen, Finland). Next, the cells were incubated with Dynal® M-450 magnetic beads coated with sheep anti-mouse IgG (Dynabeads, Dynal Biotech, Oslo), followed by magnetic separation. Separated cells were immediately studied with the ELISPOT assay.

### ELISPOT Assay of Specific ASC

The isolated PBMC and, for HR analyses, the receptor-positive and -negative cell populations were assayed for antigen-specific ASC using ELISPOT, as described earlier [13]. Briefly, the cells were incubated for two hours in 96-well microtiter plates (Maxisorp, Nunc, Roskilde, Denmark) coated in advance with antigens described above. The cells were incubated in the wells for 2 hours (2×10^6^ PBMC/mL, 50 µL/well, total 2.4×10^6^ PBMC per antigen). The antibodies secreted were detected with alkaline phosphatase-conjugated anti-human IgA (Sigma-Aldrich), IgG (Sigma-Aldrich) and IgM (SouthernBiotech, Birmingham, England). The substrate (5-bromo-4-chloro-3-indolyl phosphate p-toluidine salt; Sigma-Aldrich) was added in melted agarose. The spots were enumerated with an AID Elispot reader, and each spot was interpreted as a print of one ASC. The specificity, linearity, stability, and intermediate precision of the ELISPOT assay have been validated. A responder was defined as a person with at least 3 IgA– +IgG+IgM–ASC/10^6^ PBMC, and marked as LOD (limit of detection) of the response in the Figures. This limit has been determined over the assay validation process.

### ALS Cultures

PBMC were cultured in RPMI 1640 medium supplemented with 3 µg/mL L-glutamine (2 mM), penicillin (100 µg/mL) and streptomycin (100 µg/mL), and 10% fetal calf serum in flat-bottomed 96-well plates at 37°C in 5% CO_2_ (2×10^6^ PBMC/200 µL/well). Supernatants were collected after three days, and stored at −70°C until assayed.

### ELISA

Antibodies (IgA, IgG and IgM) in serum and ALS samples were measured with ELISA as described earlier [33]: microtiter plates (Polysorp, Nunc) were coated with a preparation of LPS of *S.* Typhi (Sigma-Aldrich; 10 µg/mL 30% methanol-PBS), and blocked with 5% milk-PBS solution. Next, the samples diluted in blocking solution were incubated in the wells overnight. The horseradish peroxidase (HRP)-conjugated rabbit anti-human IgA, IgG and IgM antibodies (all from Dako) was used as a secondary antibody, and the colour was developed with TMB peroxidase substrate (3,3',5,5'- tetramethylbenzidine and H_2_O_2_ in citric acid buffer; KPL, Gaithersburg, USA), and stopped with 0.5 M sulfuric acid. A response was defined as at least two-fold increase in the titre from the prevaccination level in the specific antibody titres.

### Statistics

Statistical analyses were carried out with JMP software, version 9.0.0 (SAS Institute Inc, Cary, NC, USA). The distribution of the ASC and HR expressions was tested with Shapiro-Wilk’s test. Since not all distributions proved normal even after log transformations, Bonferroni-corrected Wilcoxon signed rank test was used for comparisons between the responses to various antigens, and Wilcoxon signed rank test between two groups. The results are given as the means, medians, and 95% confidence intervals (CI) for the number of ASC, and as means ±95%CI for the HR expressions.

The proportions of the receptor-positive ASC were calculated as follows: percentage of receptor-positive ASC = (100× the number of ASC in receptor positive cell population)/(the total of of ASC in receptor-positive and -negative cell populations).

## Results

### ASC Response in the Vi Group

**Figure 2:**
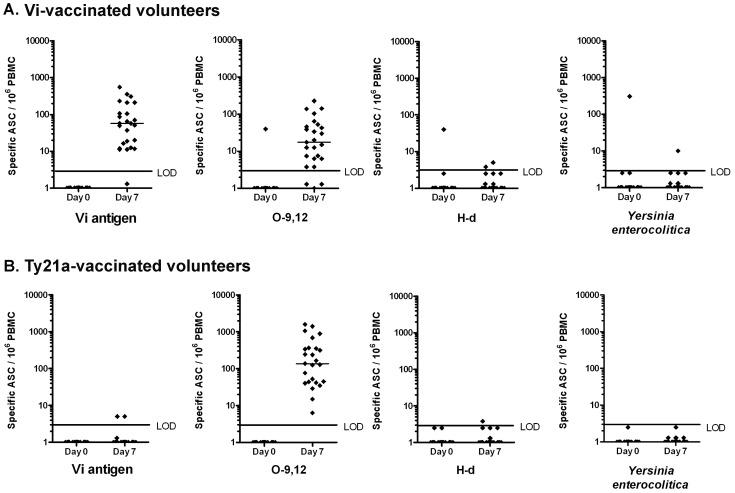
Plasmablasts on days 0 and 7 after immunization with Vi capsular polysaccharide or *Salmonella* Typhi Ty21a vaccines. Numbers of circulating plasmablasts specific to Vi, O-9,12 and H-d antigens, and to *Yersinia enterocolitica* representing a negative control. The plasmablasts were identified as antibody-secreting cells (ASC) in (a) 25 volunteers vaccinated with the parenteral Vi capsular polysaccharide vaccine and in (b) 25 age- and gender-matched volunteers receiving the oral live whole-cell *Salmonella* Typhi Ty21a vaccine lacking the Vi antigen. The dots represent results of individual vaccinees, and the lines the medians of the numbers of Ig(A+G+M)-plasmablasts on day 0 and 7 after vaccination. LOD = lower limit of detection of the response.

Before vaccination, 24 out of the 25 vaccinees had no ASC specific to any of the antigens in their circulation (Figure 2a). One volunteer had 355 ASC/10^6^ PBMC to *Salmonella* Typhi, 40 to O-9,12, 40 to H-d and 308 to *Yersinia enterocolitica*, but none to Vi antigen. As he had just one week earlier recovered from of a febrile respiratory tract infection, the ASC were interpreted as a sign of polyclonal stimulation.

**Figure 3:**
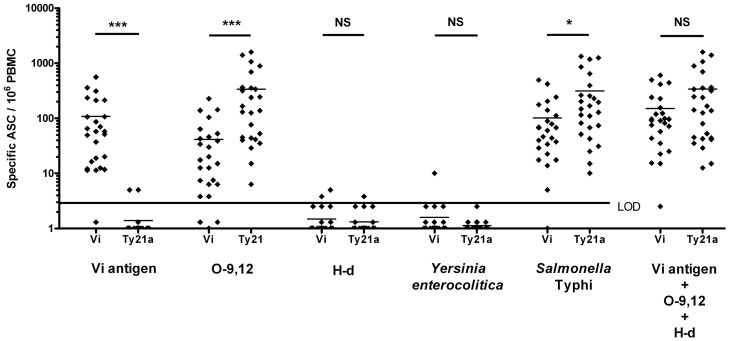
Comparison of the typhoid-specific plasmablast response in the two vaccination groups. Comparison of the numbers of circulating antigen-specific plasmablasts (ASC) between 25 volunteers immunized with the parenteral Vi vaccine and 25 with the oral Ty21a vaccine. Plasmablasts were examined for specificity against the Vi, O-9,12, and H-d antigens, against *Yersinia enterocolitica* acting as a negative control and against whole-cell *S.* Typhi. The numbers of typhoid-specific plasmablasts are also indicated as a total of cells reactive with Vi, O-9,12 and H-d antigens. The dots represent results of individual vaccinees, and the lines the means of the numbers of Ig(A+G+M)-plasmablasts on day 7 after vaccination. The statistical comparisons (Bonferroni-corrected Wilcoxon signed rank test) between the strains in the two vaccination groups are indicated with asterisks (***p<0,001; **0.001<p<0.01, *p<0,05) above the bars. LOD = lower limit of detection of the response. No LOD values are given for Vi+O-9,12+ H-d, since this does not represent a result of the assay, but a calculated total value for three different antigens.

**Figure 4:**
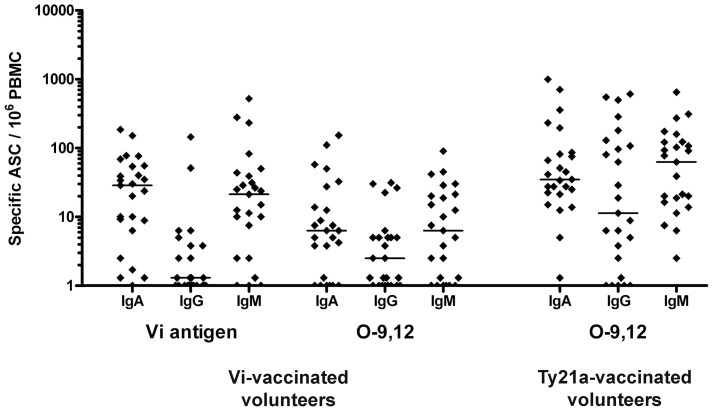
Immunoglobulin isotype distribution. Immunoglobulin isotype distribution of antibodies secreted by plasmablasts reactive with Vi and O-9,12 antigen (ASC/10^6^ PBMC) in 25 volunteers immunized with the Vi capsular polysaccharide, and with O-9,12 antigen in 25 volunteers immunized with the *Salmonella* Typhi Ty21a vaccine. The dots represent results of individual vaccinees, and the lines the medians of the numbers of plasmablasts secreting specific antibodies of the IgA, IgG or IgM isotype on day 7 after vaccination.

Seven days after vaccination, 24 out of 25 volunteers showed a response to the Vi polysaccharide and to *S.* Typhi, 22/25 to O-9,12, and 2/25 to H-d (Figure 2 and 3). The highest numbers were found with the Vi antigen and *S.* Typhi (Figure 2a, 3, Table 2), yet a substantial response was also seen to O-9,12. The responses were mainly dominated by IgA and IgM (Figure 4).

### ASC Response in the Ty21a Group

Before vaccination, none of the vaccinees had plasmablasts specific to any of the antigens. (Figure 2b). Seven days after vaccination, circulating *S.* Typhi and O-9,12 -specific ASC were detected in all vaccinees, while no response was seen to Vi antigen or H-d (Figure 2b, 3, Table 3). Here, too, the responses were mostly dominated by IgA and IgM (Figure 4).

**Table 3:** Magnitude of the plasmablast responses to various antigens and results of statistical comparisons in the Ty21a group.

Antigens and magnitude of the response in the Ty21a-vaccinated volunteers		Comparison with						
Antigen	Mean (95%CI)	Vi antigen	O-9,12	H-d	Neg. control		*S.* Typhi	
**Vi antigen**	1 (0–1)							
**O-9,12**	337 (154–520)	***						
**H-d**	1 (0–1)	NS	***					
**Neg. control**	0 (0–1)	NS	***	NS				
***S.*** ** Typhi**	314 (147–481)	***	NS	***		***		
**Sum of typhoid antigens (Vi+O-9,12+ H-d)**	339 (155–521)	***	NS	***		***		NS

The number of plasmablasts (ASC/10^6^ PBMC) reactive with the strains (means and 95% confidence intervals), and statistical comparison (Bonferroni-corrected Wilcoxon signed rank test) between the responses in 25 volunteers vaccinated with the Ty21a vaccine one week earlier. Significant differences are indicated with asterisks (***p<0.001; **0.001<p<0.01; *0.01<p<0.05), NS = not significant.

### Comparison of the ASC Responses between Vi and Ty21a Groups

Statistical comparisons between the responses in the two vaccine groups are shown in Figure 3. The response to the Vi antigen was significantly higher in the Vi group, and to O-9,12 in the Ty21a group. The total response to typhoid antigens was assessed by two approaches: as the response to whole cell *S*. Typhi, and as a total of responses to individual typhoid antigens (Vi+O-9,12+ H-d). No significant differences were seen between the groups by the latter approach.

### Expression of α_4_β_7,_ L-selectin and CLA on Specific ASC

**Figure 5:**
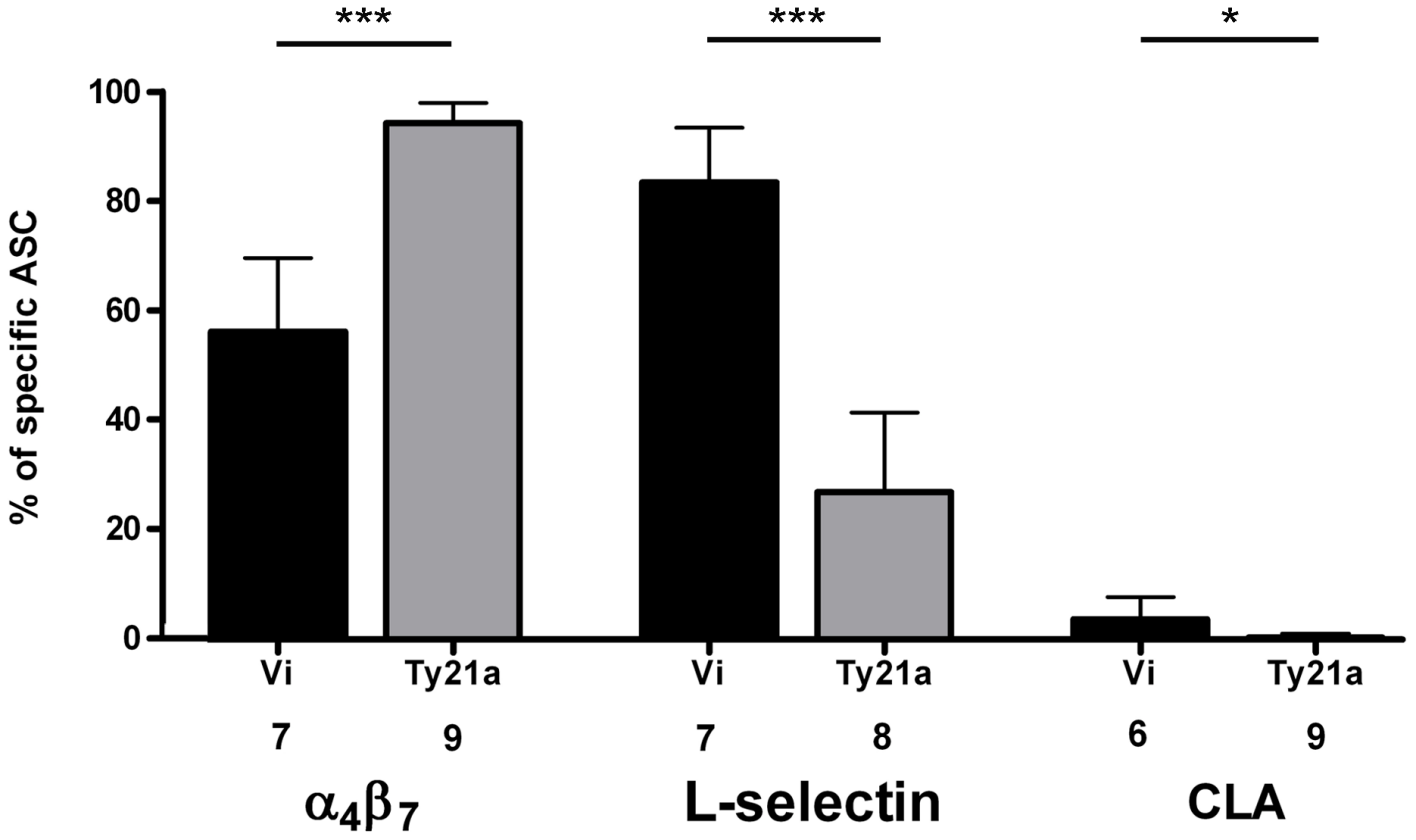
Homing potentials of *Salmonella* Typhi-specific plasmablasts in the two vaccination groups. The expression of α_4_β_7_, L-selectin and CLA on *Salmonella* Typhi-specific plasmablasts in the peripheral blood of volunteers seven days after vaccination with the Vi or the Ty21a vaccine. The bars indicate the arithmetic means +95%CI of percentages of HR-positive ASC among all pathogen-specific ASC (IgA+IgG+IgM). The HRs and the numbers of volunteers from whom the data were pooled are indicated under the data bars. The data on HR expressions on *S*. Typhi-specific plasmablasts in Ty21a-vaccinated volunteers has been reported earlier [32]. The statistical comparisons (Wilcoxon signed rank test) between the strains in the two vaccination groups are indicated with asterisks (***p<0,001; **0.001<p<0.01, *p<0,05).

The numbers of PBMC obtained only allowed a HR analysis on plasmablasts specific to one antigen. The whole-cell *S.* Typhi was chosen for this. In the Vi group, a systemic homing profile was seen: 84% of all *S.* Typhi-specific ASC expressed the peripheral lymph node HR, L-selectin, and only 56% the intestinal HR, α_4_β_7_-integrin, and 4% the cutaneous HR, CLA. In the Ty21a group the HR profile was mucosal, as evidenced by the very high expression of α_4_β_7_ (95%) and lower expression of L-selectin (27%) (Figure 5); these data on HR expressions on *S*. Typhi-specific plasmablasts in Ty21a-vaccinated volunteers were retrieved from our previous report [32] to allow comparison between the two vaccine groups. The comparison to the HR-profile in natural typhoid fever (α_4_β_7_ 91%, L-selectin 40%, CLA 11%) obtained from the data base of our previous study [32] showed that only the response in the Ty21a group imitated that in a natural infection. The case of relapsed typhoid fever had a unique profile with a high expression of all HR (α_4_β_7_ 95%, L-selectin 66%, CLA 65%).

### Antibodies in ALS and Serum Samples

The number of vaccinees with a twofold or higher rise in antibody titres in serum and ALS samples was smaller than of those responding in the ELISPOT assay (Table 4).

**Table 4:** Antibody responses in serum and ALS cultures.

Assay	Vi vaccine				Ty21a vaccine			
	IgA	IgG	IgM	IgA/IgG/IgM	IgA	IgG	IgM	IgA/IgG/IgM
**Serum antibody** ELISA Vi: n = 25 Ty21a: n = 25	4	4	4	8 (32%)	7	5	12	13 (52%)
**ALS** ELISA Vi: n = 11 Ty21a: n = 12	1	0	1	1 (9%)	11	4	10	11 (84%)
**ELISPOT** Vi: n = 25 Ty21a: n = 25	22	19	22	24 (96%)	24	18	24	25 (100%)

Numbers of vaccinees responding in serum, ALS culture and ELISPOT assays for *Salmonella* Typhi in the Vi and Ty21a groups. In the ELISA assays (serum and ALS), a responder was defined as an individual with at least two-fold increase in titre (in IgA, IgG and/or IgM isotype). In the ELISPOT assay, a responder was defined as having at least 3 ASC/10^6^ PBMC on day 7. Blood samples for ELISPOT and ALS analyses were collected on days 0 and 7, and serum samples on days 0 and 28 after vaccination. The number of vaccinees tested with each assay is indicated in the left row.

## Discussion

The present study is the first head-to-head study comparing immune responses to Vi and Ty21a vaccines, the two typhoid vaccines currently available. We found similar numbers of typhoid-specific plasmablasts in the circulation of the vaccinees in both groups, yet the homing profiles were found to be different. As an unforeseen finding, the antigen-specificity of these responses proved less different than had been thought.

Until now, no studies have explored circulating Vi-specific plasmablasts in volunteers receiving typhoid vaccines. A clear Vi-specific response was mounted in the Vi group, while none in the Ty21a group responded to this antigen, in alignment with the lack of Vi antigen in the Ty21a strain. In the Ty21a group, the highest response was mounted to O-9,12 antigen. No response to O-9,12 was initially anticipated in the Vi group, yet O-antigen-specific plasmablasts were found in almost all vaccinees. Even if significantly lower than in the Ty21a group, the response was clear.

The response to the typhoidal O-antigen in the Vi group calls for an explanation. A logical reasoning derives from the fact that the Vi polysaccharide was initially isolated from the *Salmonella* Typhi Ty2 strain. Since it is not an easy task to remove the contaminating lipopolysaccharide (LPS), traces of typhoidal LPS are likely to be left in the final preparation. This amount appears small enough not to cause adverse effects typical to parenteral whole-cell typhoid vaccines, yet high enough for the immune system to recognize it. In fact, in early studies, serum antibodies to LPS had been reported in Vi vaccinees, the levels correlating positively to the LPS content of the preparation: 5% of LPS caused seroconversion in 83% of vaccinees, and 0.2% of LPS in only 26% [9].

In the present study, no immune response to H-d antigen was observed. In our previous study, a plasmablast response against H-d antigen was found after parenteral whole-cell Ty21a, but not after oral Ty21a vaccination [19], suggesting immunization route-dependent differences in processing the antigen. In the present study, the lack of response to H-d found in the Vi group suggests that either the antigen has been destroyed in the vaccine purification process, or the amount that is left of it remains too low to elicit a measurable response.

Previous studies with Ty21a vaccines have shown that the numbers of plasmablasts 7 days after vaccination correlate with levels of effectiveness observed in field trials using the same immunization regimen [10], [13]. As the Vi and Ty21a vaccines have in various field trials been reported to confer a similar degree of protection, we were curious to know whether they elicit similar levels of typhoid-specific plasmablasts in the circulation. The total typhoid-specific response was estimated with two separate approaches: by using whole-cell *S.* Typhi as antigen in the assay, and by calculating the total of plasmablast responses to all typhoidal antigens measured, i.e. to Vi, O-9,12, and H-d antigens. In the Ty21a group these two approaches yielded identical results, whereas in the Vi group the former approach appeared less sensitive than the latter. We suggest that using whole-cell *S.* Typhi as an antigen may not allow a sufficiently high concentration of the Vi antigen in the assay or, alternatively, the Vi polysaccharide may have been partly destroyed by formalin, when preparing the strain for the assay. Therefore, the response to Vi antigen should be assessed separately, as was done in the latter approach. The total of the response to the different typhoidal antigens (Vi+O-9,12+ H-d) was found to be similar in the two vaccine groups, consistent with the similar efficacy of these vaccines in field trials [2]–[4]. Interestingly, the magnitude of the response in a natural typhoid infection (for three patients mean specific ASC/10^6^ PBMC against *S*. Typhi 343, O-9,12 216, H-d 3 and *Yersinia enterocolitica* 3) in our previous studies [32], [33] appears similar to that reported for the two vaccine groups in the present study.

Immunity to typhoid fever is considered to consist of several factors, including both cell-mediated and humoral immune mechanisms [13]–[19]. The conception that the immune mechanisms of these two vaccines are totally different would imply that typhoid immunity can be attained in at least two entirely different ways: through antibodies against Vi antigen (as with Vi vaccine) or by several other mechanisms, e.g. by eliciting cell-mediated immune responses, by specific intestinal secretory IgA, and LPS-specific serum antibodies (Ty21a vaccine). However, an O-antigen-specific plasmablast response also conferred by the Vi vaccine suggests that the current conception of the protective mechanisms of this vaccine may need to be revisited. O-antigens are considered to contribute to the defence conferred by whole-cell parenteral vaccines: protective efficacy has been reported by vaccines prepared both from Vi-positive (K strain) and -negative (L strain) *S.* Typhi strains [34]. Interestingly, *Citrobacter* 5396/38 and *S.* Typhi have identical Vi antigens. In early studies, Vi polysaccharide isolated from the *Citrobacter* strain was found to be less effective than the one isolated from *S.* Typhi [35]. The authors wondered whether the isolation of Vi antigen had been less successful from the *Citrobacter* than *S*. Typhi Ty2 strain. One might also argue that only the latter preparation contained typhoidal LPS. Similarly, it thus appears possible that the protection conferred by the current Vi vaccines, while mainly depending on Vi-specific mechanisms, could also include mechanisms specific to the O-antigen part.

The homing profile of the plasmablasts reflects the expected localization of the immune effector cells in the body. The plasmablasts in the Vi group exhibited a systemic homing profile, with a high frequency of L-selectin- and a lower frequency of α_4_β_7_- and CLA-expressing cells. In the Ty21a group, by contrast, the homing profile was intestinal [32], with all cells expressing α_4_β_7,_ and a lower proportion L-selectin and CLA. These results are consistent with previous studies on oral [23], [25]–[29] and parenteral [25], [27] antigen administration showing that the expression of HR depends on the site of antigen encounter [25], [27]. Both intestinal and systemic immune defence is needed in the protection against typhoid fever. The intestinal targeting appears particularly beneficial from the perspective that the pathogen uses this site as a portal of entry, a fact reflected as a gut-directed HR profile in patients with a natural typhoid infection. These data complement the view that in contrast to Vi, vaccination with Ty21a closely imitates the natural typhoid infection.

Serum antibodies are the classical approach to studying humoral immune responses. While serum antibodies provide information on the systemic immune response, the plasmablast assays (ELISPOT and ALS) describe the total response elicited in the body. Consistent with previous studies [13], [16], [33], the ELISPOT assay assessing the response at single-cell level proved to be the most sensitive approach to the evaluation of the response.

Consistent with the level of protection reported in field trials, the magnitude of the typhoid-specific plasmablast responses to Vi and Ty21a vaccines are similar. However, there is a difference in the expected localization of the response, and the antigenic specificity. Only the response to Ty21a vaccination was found to imitate closely that in a natural infection. Surprisingly, the antigen specificity assays also revealed a significant O-antigen-specific response in the Vi group, presumably caused by trace amounts of lipopolysaccharide remaining in the vaccine preparation after purification. It remains possible that part of the immunity conferred by the Vi vaccine may, in fact, be contributed by a response to typhoidal O-antigens.

## Supporting Information

Checklist S1 CONSORT checklist.
(DOC)

Protocol S1 Study protocol.
(DOC)
