# Supplementary material for: Head-to-Head Comparison of Humoral Immune Responses to Vi Capsular Polysaccharide and Salmonella Typhi Ty21a Typhoid Vaccines–A Randomized Trial
Source: PLoS One. 2013 Apr 8;8(4):e60583. doi: 10.1371/journal.pone.0060583 (PMC3620468; doi:10.1371/journal.pone.0060583)
Supplement: Protocol S1 — Study protocol. (DOC) [file pone.0060583.s002.doc]

**STUDY PROTOCOL**

**SYNOPSIS**

Ty21a - ASC

Cross-reactive immunity elicited by oral and parenteral typhoid vaccines against non-typhoid Salmonellae

| **Version** | **Date** |
| --- | --- |
| Version 2.0 (final) | 15-November-2009 |
|  |  |
|  |  |

Study code

Ty21a - ASC

**Title of the study**Cross-reactive immunity elicited by oral and parenteral typhoid vaccines against non-typhoid Salmonellae

**EudraCT registration**Number: 2009-012949-33

Clinical phase

Phase IV

Indication and rationale

Many Salmonella spp causing gastroenteritis share O antigen serotypes with *Salmonella enteritidis* subsp. *enterica* serovar Typhi (*S.typhi*) and could therefore be in ‚reach‘ of the protective efficacy of the oral live typhoid fever vaccine Vivotif®. Some of these Salmonellae are common causes of diarrhoea in travellers (0-30% of travellers diarrhea depending on the area). In a controlled study in healthy adults the cross-reactivity of the immune response against different Salmonella spp. elicited by Vivotif® shall, therefore, be investigated and compared to the immune response of Typherix®

Study design

Randomized (1:1, Vivotif® vs. Typherix®), controlled, mono-center study

Study objectives

*Primary:*  - Demonstration of cross-reactive antibodies against O9 and/or O12 somatic antigen
 containing Salmonella spp after immunisation with Vivotif (in comparison to
 Typherix®)

*Secondary:*  - To evaluate the homing potentials of circulating vaccine antigen-specific antibody-
 secreting cells towards gut mucosa, systemic immune system and cutaneous sites
 after immunisation with Vivotif® (in comparison to Typherix®)
 - To evaluate the immunogenicity of the typhoid fever vaccine Vivotif® (in comparison
 to Typherix®) in terms of the agglutination antibody response (Widal) and

Salmonella-specific antibody response in serum, saliva and tears (ELISA)

- To analyse the cell-mediated immune response to S.typhi and selected Salmonella

spp (prolifieration assay and IFN-gamma production)

Investigator and study center

Anu Kantele MD PhD
Helsinki University Central Hospital, Helsinki, Finland

Study duration planned

Recruitment period: 11 months

First subject first visit: December 2009

Last subject last visit: October 2010

Treatments
The investigational medicinal products (IMP's) are:
Group A - ***Vivotif:* three oral doses administered with an interval of 1 day (Day 0 [Visit 1] and two self-administrations at home on Days 2 and 4). One dose of** Vivotif® (enteric-coated capsule) contains:

- A total of ≥2x10^9^ live *S.typhi* Ty21a bacteria/capsule

Group B - *Typherix***®***:* one intramuscular dose will be administered at Visit 1 (Day 0). One dose (0.5 mL) in a ready-to-use syringe contains:

- 25 mcg of Vi capsular polysaccharide of *S.typhi* Ty2

Number of subjects

50 randomized subjects

**Study Population**

Healthy adults aged ≥18 to ≤65 years (n = 50)

Inclusion criteria

1. Male or female subjects aged ≥18 to ≤65 years
2. General good health as established by medical history and physical examination
3. Written informed consent
4. Females of childbearing potential must agree to use an efficacious hormonal or barrier method of birth control during the study. Abstinence is acceptable.
5. Available for all visits scheduled in this study.

Exclusion criteria

1. Vaccination against typhoid fever within 5 years before dosing.
2. History of clinical typhoid fever, clinical paratyphoid A or B fever.
3. Immunization with any other vaccine (oral or parenteral) within 4 weeks prior to study start or planned vaccination during the study
4. Current intake of antibiotics or end of antibiotic therapy <8 days before first IMP administration
5. Chronic (longer than 14 days) administration of immunosuppressants or other immune-modifying drugs within 6 months before the first dose of IMP; oral corticosteroids in dosages of ≥0.5 mg/kg/d prednisolone or equivalent are excluded; inhaled or topical steroids are allowed
6. Acute or chronic clinically significant gastrointestinal disease
7. Any confirmed or suspected immunosuppressive or immunodeficient condition, including human immunodeficiency virus (HIV) infection
8. Pregnancy or lactation
9. Acute disease at the time of enrollment (defined as the presence of a moderate or severe illness with or without fever (fever is defined as body temperature of ≥38 °C).
10. Alcohol or drug abuse
11. Suspected non-compliance
12. Use of any investigational drug or any investigational vaccine within 30 days preceding the first dose of study vaccine, or planned use during the study period
13. Any clinically significant history of known or suspected anaphylaxis or hypersensitivity reaction based on the judgement of the investigator
14. Employee at the investigational site, relative or spouse of the investigator
15. Any other criteria which, in the investigator’s opinion, would compromise the ability of the subject to participate in the study, the subject’s well-being, or the outcome of the study

Criteria for evaluation

*Primary endpoint:*

- Measurement of specific IgA -, IgG- and IgM-secreting antibody cells specific to / cross-reactive with the different Salmonella serovars in peripheral blood using ELISPOT

*Secondary endpoints:*

- To evaluate the expression of various homing receptors (intestinal, systemic, cutaneous) on antigen-specific antibody-secreting cells by separation the PBMCs with immunomagnetic cell sorting according to their HR expressions and by investigating specific ASC in the resulting subpopulations
- Anti-Salmonella spp. agglutination antibody response in serum (Widal test)
- Anti-Salmonella antibodies in serum, saliva or tears (ELISA)

Procedures

- Screening for eligibility (inclusion/exclusion criteria)
- Gender stratification
- Randomization 1:1 to receive Vivotif**®** (Group A) or Typherix**®** (Group B)
- 1^st^ Blood sampling prior to 1^st^ administration of IMP on Day 0 (Visit 1)
- Immunization on Days 0, 2 and 4 with Vivotif**®** and on Day 0 with Typherix**®**, respectively
- Recording of unsolicited AEs and of SAE's throughout the whole study
- 2^nd^ Blood sampling 7 days after administration of 1^st^ dose of Vivotif**®** and of the single dose of Typherix**®** ,i.e. on Day 7 for group A and group B, respectively
- Telefone contact on Day 26±1 to enquire on any intercurrent AE and remind the subject re Visit 3 (Day 28±3)
- 3^rd^ Blood sampling 4 weeks (Day 28±3) after study start

AEs and SAEs will be reviewed, documented and reported up to Visit 3 (Day 28 ± 3). Subjects will be instructed to report any SAEs perceived during 6 months after the last test IMP administration.

Contraindications will be checked for every subject before vaccination. Females of childbearing potential must make an oral statement confirming that they are not pregnant and they will be instructed to report a pregnancy occurring/becoming known during the study period (these will have to be followed-up until birth and the outcome reported to Berna Biotech-Crucell)

**Statistical methods***Sample size*

No sample size calculation has been performed. The group size is considered to be sufficient to detect and document the difference in cross-reactivity of the immune response between Vivotif**®** and Typherix**®**.

A total of 50 subjects will be randomized in a 1:1 ratio to Vivotif**®** or Typherix**®**.

Categorical data will be presented in contingency tables along with frequencies and percentages. Continuous data will be summarized using at least the frequency (n), mean, standard deviation, median and the range. Descriptive analyses for demographic baseline characteristics will be presented for all subjects and by treatment group for the total population and the subsets.

Adverse events (AEs will be presented using descriptive statistics (incidence rates). Any serious adverse events (SAEs) and discontinuations due to adverse events will be described in detail. In addition, all data will be reported in subject listings.

The results of antibody secreting cells (ASCs) will be given as ASC / 10^6^ PBMC. These data are used to calculate geometric means ± SEM; the two groups will be compared with each other with Student’s t-test.

The expression of homing receptors on ASC will be documented as percentages of ASC expressing the given marker. These data are used to calculate arithmetic means ± SD; the two groups will be compared with each other with Student’s t-test.

The geometric mean titers (GMTs) and corresponding 95% CIs of the Widal agglutination antibodies will be presented together with a descriptive summary (mean, standard deviation [s.d.]) of the original antibody values by study group and visit.

**Figure 1: Schedule of assessments**

|  | **Visit 1** Baseline |  |  | **Visit 2** | **Visit 3** |
| --- | --- | --- | --- | --- | --- |
| Timing | Day 0 | Day 2 | Day 4 | Day 7 | Day 28 ± 3  *(Days 25 to 31)* |
| Informed consent | X |  |  |  |  |
| Inclusion/exclusion criteria | X |  |  |  |  |
| Demography and medical history | X |  |  |  |  |
| Physical examination | X |  |  |  |  |
| Contraindications ^a^ | X |  |  |  |  |
| Gender stratification / Randomization | X |  |  |  |  |
| Blood sampling | X |  |  | X | X |
| Vivotif**®** /Typherix**®** administration | X^b^ | X^c^ | X^c^ |  |  |
| Adverse events ^d^ | X |  |  | X |  |
| Concomitant medication | X |  |  | X |  |

^a^ To be performed pre-dose.
^b^ Subjects will be closely observed for 20 min after the 1^st^ IMP administration.

^c^ Subjects in the Vivotif**®** group will self-administer the vaccine at home.

^d^ Subjects are instructed to report any SAEs perceived during 6 months after last IMP administration.

**References**- Kantele A et al. Specific immunoglobulin-secreting human blood cells after peroral vaccination
 against Salmonella typhi. J Infect Dis 1986; 153: 1126-31

- Kantele A: Antibody secreting cells in the evaluation of the immunogenicity of an oral vaccine,
 Vaccine 1990;8:321-6

- Kantele A, Arvilommi H, Kantele JM, Rintala L, Mäkelä PH: Comparison of the human immune
 response to live oral, killed oral or killed parenteral *Salmonella typhi* Ty21a vaccines, Microbial
 Pathogenesis 1991;10:117-26

- Kantele A, Kantele JM, Savilahti E, Westerholm M, Arvilommi H, Lazarovits A, Butcher EC, Mäkelä
 PH: Homing potentials of circulating lymphocytes in humans depend on the site of activation: oral,
 but not parenteral, typhoid vaccination induces circulating antibody-secreting cells that all bear
 homing receptors directing them to the gut, J Immunol 1997;158:574-9

- Kantele A et al. Unique characteristics of intestinal immune system as inductive site after antigen
 re-encounter. J Infect Dis 2005; 191: 312-317
